# Rationale and methods of the European Study on Cardiovascular Risk Prevention and Management in Daily Practice (EURIKA)

**DOI:** 10.1186/1471-2458-10-382

**Published:** 2010-06-30

**Authors:** Fernando Rodríguez-Artalejo, Eliseo Guallar, Claudio Borghi, Jean Dallongeville, Guy De Backer, Julian P Halcox, Ramón Hernández-Vecino, Francisco Javier Jiménez, Elvira L Massó-González, Joep Perk, Philippe Gabriel Steg, José R Banegas

**Affiliations:** 1 Department of Preventive Medicine and Public Health. School of Medicine. Universidad Autónoma de Madrid. CIBER of Epidemiology and Public Health. Madrid 28029, Spain; 2 Departments of Epidemiology and Medicine and Welch Center for Prevention, Epidemiology, and Clinical Research. Johns Hopkins Bloomberg School of Public Health. Baltimore, MD, 21205, USA; 3 Department of Cardiovascular Epidemiology and Population Genetics. National Center for Cardiovascular Research (CNIC). Madrid 28029, Spain; 4 Department of Internal Medicine, Aging and Clinical Nephrology. University of Bologna, Bologna, Italy; 5 Inserm U 744, Institut Pasteur de Lille. 59019 Lille Cedex, France; 6 Department of Public Health. University of Gent, 9000 Gent, Belgium; 7 Wales Heart Research Institute, Cardiff University, Heath Park, Cardiff CF14 4XN, UK; 8 Medical Department. AstraZeneca Farmacéutica Spain, S.A, 28003 Madrid; 9 Medical Department, AstraZeneca Europe, 1935 Zaventem, Belgium; 10 School of Health and Caring Sciences, Linnaeus University, 391 82 Kalmar, Sweden; 11 INSERM U 698, Assistance Publique-Hôpitaux de Paris and Université Paris 7, Paris

## Abstract

**Background:**

The EURIKA study aims to assess the status of primary prevention of cardiovascular disease (CVD) across Europe. Specifically, it will determine the degree of control of cardiovascular risk factors in current clinical practice in relation to the European guidelines on cardiovascular prevention. It will also assess physicians' knowledge and attitudes about CVD prevention as well as the barriers impeding effective risk factor management in clinical practice.

**Methods/Design:**

Cross-sectional study conducted simultaneously in 12 countries across Europe. The study has two components: firstly at the physician level, assessing eight hundred and nine primary care and specialist physicians with a daily practice in CVD prevention. A physician specific questionnaire captures information regarding physician demographics, practice settings, cardiovascular prevention beliefs and management. Secondly at the patient level, including 7641 patients aged 50 years or older, free of clinical CVD and with at least one classical risk factor, enrolled by the participating physicians. A patient-specific questionnaire captures information from clinical records and patient interview regarding sociodemographic data, CVD risk factors, and current medications. Finally, each patient provides a fasting blood sample, which is sent to a central laboratory for measuring serum lipids, apolipoproteins, hemoglobin-A1c, and inflammatory biomarkers.

**Discussion:**

Primary prevention of CVD is an extremely important clinical issue, with preventable circulatory diseases remaining the leading cause of major disease burden. The EURIKA study will provide key information to assess effectiveness of and attitudes toward primary prevention of CVD in Europe. A transnational study creates opportunities for benchmarking good clinical practice across countries and improving outcomes. (ClinicalTrials.gov number, NCT00882336.)

## Background

Despite important progress in prevention and treatment, the epidemic of cardiovascular disease (CVD) in Europe is far from controlled. With over 4.3 million deaths per year, CVD is the foremost cause of death across Europe and the leading cause of disability adjusted life years [1].

Both CVD mortality and its trends vary within Europe. The incidence of and mortality from coronary heart disease (CHD) is declining in various countries in Northern and Central Europe, where the risk of CVD has been highest for several decades. In contrast, CHD mortality has been falling less rapidly, and is even increasing in Eastern Europe. In the Mediterranean area, the recent decline in morbidity has been more moderate, although from a background of lower mortality [1]. In these countries, the growth and progressive ageing of the population in recent years have led to the paradoxical situation of reduced age-adjusted CHD mortality on the background of an increased number of deaths which tend to occur later in life and, consequently, a greater burden of disease and disability [2]. This paradoxical increase in the population burden of CHD is emerging even in countries with marked reductions in age-adjusted CHD mortality, such as England and Wales [3].

There is a large and compelling body of evidence on the efficacy of primary CVD prevention [4], and of its population impact. For example, in several European countries most of the reduction in CHD mortality in recent decades has been due to interventions focusing on reducing the impact of classic CVD risk factors, at the population level or in individual patients in clinical practice [5-7]. However, the vast residual burden of CVD suggests that there is an important unmet need for primary CVD prevention in Europe.

### Primary cardiovascular prevention in clinical practice in Europe

The majority of information available on the management of risk factors for CVD in clinical practice is limited to patients with established CVD or with very high CVD risk such as those with diabetes mellitus. Several studies such as the REACH registry or EUROASPIRE have shown a high prevalence of undertreated CVD risk factors (particularly those related to lifestyle) among outpatients and hospitalized patients with atherothrombosis [8,9]. It is both notable and disappointing that, despite the advances in the evidence base, global risk factor control seems to have improved little in these high risk patients since 1995 [10].

The EPA Cardio project is evaluating the quality of CVD prevention in primary care in 10 European countries; however, it only studies higher risk patients (over 10% risk of death from CVD in 10 years or at least 3 CVD risk factors) [11]. Thus, there is little information in Europe on the clinical management of CVD risk factors across the whole spectrum of primary prevention. In particular, it is not known whether patients are being managed in accordance with recent European guidelines on CVD prevention [4]. A central element in these guidelines is the measurement of global CVD risk according to the SCORE equation [12], and adjustment of the intensity of the intervention to the magnitude of the risk. Although a previous edition of the European guidelines recommended calculation of CVD risk with the SCORE [13], there has been no comprehensive assessment of the extent of use of formal risk assessment systems, the selection of risk assessment tools and the use of such estimates of risk by European physicians in clinical decision-making.

A better understanding of these issues is key for designing interventions to overcome the barriers preventing implementation of the European guidelines on CVD prevention.

### Barriers impeding the use of global cardiovascular risk and the adoption of other recommendations from the European guidelines for CVD prevention

Just over 10 years ago, Cabana and colleagues proposed a model to explain the reasons why physicians do not follow clinical practice guidelines [14]. These reasons included lack of awareness of existing guidelines, lack of familiarity with them, disagreement with some aspect of the guidelines, lack of self-efficacy performing a certain behavior, or the presence of external barriers such as limited resources (time, physical space, reimbursement for procedures, etc.)

Some studies in Europe have examined physicians' knowledge and attitudes about CVD prevention, especially in primary care. However, these studies were often small [15,16], focused on control of only a single risk factor such as dyslipidaemia [17], or provided data limited to a single country [18]. Thus, in contrast to the knowledge available in the United States of America (USA) [19,20], there is no comprehensive European picture of the barriers impeding effective control of CVD risk factors and reducing adherence to the recommendations of European guidelines for primary CVD prevention. However, the rich mix within Europe in the organization of National health care systems, in material resources, in academic training of physicians and in patients' cultural and socioeconomic backgrounds provides an opportunity to learn about the most favorable conditions for primary CVD prevention. Although many of the existing barriers are likely to operate at the local level and are likely to require more local solutions, a standardized, contemporaneous trans-national comparison creates an opportunity to emulate those clinical practices that work well in other countries.

### The role of new biomarkers in dyslipidaemia management

Improved reduction of cholesterol levels in persons free from CVD is one of the factors that has contributed most to the decline in CHD mortality in some European countries [5,6] and in the USA [21]. Consequently, it seems reasonable to optimize control of cholesterol to reduce residual CVD risk, with low density lipoprotein (LDL) cholesterol the principal therapeutic target in lipid management. However, a recent practice guideline suggests the use of alternative biomarkers, such as non high density lipoprotein (HDL) cholesterol (total cholesterol minus HDL cholesterol) or apolipoprotein-B (apo B) to monitor the effects of lipid-lowering treatment, especially in patients who have already reached low or moderate levels of LDL cholesterol but also have various other cardiometabolic risk factors [22], particularly elevated triglycerides. Unlike LDL cholesterol, measurement of non-HDL cholesterol and apo B does not require overnight fasting. In addition, both biomarkers are better predictors of CVD risk than LDL cholesterol, especially in patients treated with statins [23,24]. Therapeutic goals have recently been proposed for non-HDL cholesterol and apo B [22]. However, data are lacking on the effectiveness of dyslipidaemia management based on these biomarkers, and thus on the opportunities therefrom for reducing the residual CVD risk associated with dyslipidaemia.

In addition, chronic low grade inflammation is known to contribute to the development and progression of atherothrombosis; furthermore, one marker of inflammation, high sensitivity C-reactive protein (hs-CRP), is a modest but consistent predictor of CVD events [25]. The recent JUPITER study has shown that rosuvastatin reduces major CVD events and all-cause mortality in patients without elevated LDL cholesterol but with modestly elevated hs-CRP [26]. This is important because many people who develop acute myocardial infarction or stroke are apparently healthy and have cholesterol levels and global risk scores below the thresholds for more intensive preventive treatment.

Most trans-national studies on the clinical management of dyslipidaemia in primary CVD prevention have been based on medical record reviews and on interviews with physicians and patients [11,15,17,27], but lacked contemporary biological data such as fasting blood samples. Thus, previous studies in this area have been unable to assess levels and model the impact of emerging biomarkers such as non-HDL cholesterol, apo B and hs-CRP, since these tests were not routinely performed.

### Study objectives

The European Study on Cardiovascular Risk Prevention and Management in Usual Daily Practice (EURIKA) is an international research initiative conducted in 12 European countries. Its overall aim is to assess the status of primary CVD prevention in the clinical setting across Europe. The specific aims are to determine the degree of CVD risk factor control according to the updated European guidelines on CVD prevention [4], and to identify the systems used and barriers faced by physicians in controlling CVD risk factors as well as their attitudes towards this aspect of their current clinical practice. Blood samples are also collected from EURIKA study participants to measure and explore the role of novel biomarkers for identification of patients at increased risk of CVD who might benefit from more intensive interventions.

## Methods/Design

### Design

**Figure 1:**
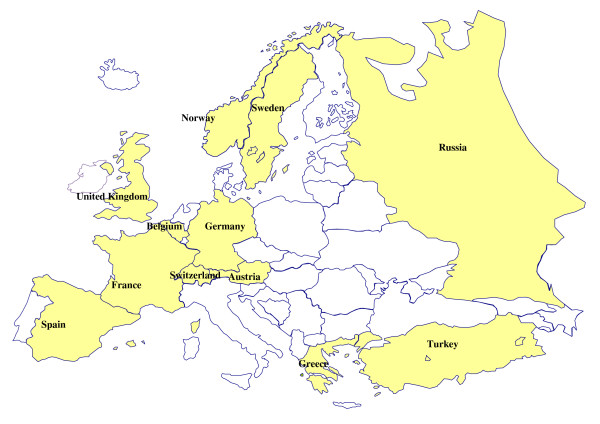
**Countries participating in the EURIKA study**.

The EURIKA study is a multinational, cross-sectional study conducted in 12 countries (Austria, Belgium, France, Germany, Greece, Norway, Russia, Spain, Sweden, Switzerland, Turkey, and the United Kingdom) (**figure **1). These countries were selected to represent the whole spectrum of CVD risk, risk factor control, and organization of health-care services across Europe. Data collection started in May 2009 and was completed in January 2010. All participating patients provided signed informed consent The study protocol has been approved by the appropriate clinical research ethics committees in each participating country, and complies with the local regulations for clinical research. In particular the protocol was approved by the following ethics committees: Ethics Committee of Hospital Barmherzige Brüder, Vienna, Austria; Ethics Committee University Hospital, Ghent. Belgium; National Commission on Informatics and Liberties, Paris, France; Ethics Committee of the Friedrich-Alexander-University Erlangen-Nuremberg, Germany; Scientific Council of University General Hospital of Ioannina, and the National Organization for Medicines - EOF), Greece; Regional Committee for Ethics in Medicine and Research Sor-∅st B (REK Sor-∅st B, Oslo, Norway; Independent Interdisciplinary Ethics Committee, Moscow, Russia; Clinical Research Ethics Committee of La Paz University Hospital, Madrid, Spain; Ethics Committee of the University hospital of Linköping, Sweden; Ethics Committee for Ambulatory Clinical Research. Medical Association of Geneva, Switzerland; Research Ethics Committee of Medical Faculty, Gazi University, Ankara, Turkey; Brent Primary Care Trust applied research unit, National Health Service, London, UK

### Study participants

The study sample was selected in a two-step process that involved recruitment of physicians and their outpatients. Physicians were selected to represent current practitioners in primary care centers or outpatient clinics involved in CVD prevention in each country. Rosters of practicing physicians in each country were obtained from the OneKey database, a large database containing information on the characteristics of practicing physicians (table 1) http://www.cegedim.com/corporate/cegedim_eng/cegedimdendrite.htm. This database was used to select a random sample of physicians stratified by age, sex and specialty, including family medicine and other medical specialties involved in CVD risk factor control, such as cardiology, internal medicine, and endocrinology. Physician sex and age strata are proportional to their distribution in the OneKey database. To determine the proportion of practitioners in each medical specialty invited to participate, we followed the advice of key practicing physicians in each country who were interviewed about local characteristics of the health care system and the participation of each type of medical specialist in CVD prevention. Based on their responses, the proportion of physicians in each specialty varies across countries, although family physicians working at the primary care level predominate in all countries. The EURIKA study includes approximately 60 physicians per country (table 2). The percentage of participating physicians among those invited is 7.9% for the whole study (range 3.1% in Sweden to 22.8% in Turkey).

**Table 1:** Physicians* included in the oneKey database, by sex and country

		Sex	
		
	Total	Men	Women
	N	N (%)	N (%)
**Austria**	9848	5772 (58.6)	4076 (41.4)
**Belgium**	12588	8527 (67.7)	4061 (32.3)
**France**	69173	49448 (71.5)	19725 (28.5)
**Germany**	74963	47740 (63.7)	27223 (36.3)
**Greece**	11699	8131 (69.5)	3568 (30.5)
**Norway**	6181	3931 (63.6)	2250 (36.4)
**Russia**	54592	6798 (12.5)	47794 (87.5)
**Spain**	69266	31726 (53.5)	27540 (46.5)
**Sweden**	8740	5099 (58.3)	3641 (41.7)
**Switzerland**	8093	5980 (73.9)	2113 (26.1)
**Turkey**	39825	27053 (67.9)	12772 (32.1)
**United Kingdom**	44330	26085 (58.8)	18245 (41.2)
**Total**	399298	226290 (56.7)	173008 (43.3)

*General medicine/family medicine, internal medicine, cardiology and endocrinology.

**Table 2:** Physicians* participating in the EURIKA study, by sex, age and country

		Sex		Age	
			
	Total	Men	Women	< 50 years	≥ 50 years
	N	N (%)	N (%)	N (%)	N (%)
**Austria**	62	40 (64.5)	22 (35.5)	22 (35.5)	40 (64.5)
**Belgium**	78	57 (73.1)	21 (26.9)	56 (71.8)	22 (28.2)
**France**	55	37 (67.3)	18 (32.7)	18 (32.7)	37 (67.3)
**Germany**	66	48 (72.7)	18 (27.3)	25 (37.9)	41 (62.1)
**Greece**	63	41 (65.1)	22 (34.9)	45 (71.4)	18 (28.6)
**Norway**	57	39 (68.4)	18 (31.6)	41 (71.9)	16 (28.1)
**Russia**	93	13 (14.0)	80 (86.0)	64 (68.8)	29 (31.2)
**Spain**	70	37 (52.9)	33 (47.1)	35 (50.0)	35 (50.0)
**Sweden**	57	42 (73.7)	15 (26.3)	16 (28.1)	41 (71.9)
**Switzerland**	71	55 (77.5)	16 (22.5)	39 (54.9)	32 (45.1)
**Turkey**	68	55 (80.9)	13 (19.1)	57 (83.8)	11 (16.2)
**United Kingdom**	69	48 (69.6)	21 (30.4)	44 (63.8)	25 (36.2)
**Total**	809	512 (63.3)	297 (36.7)	462 (57.1)	347 (42.9)

*General medicine/family medicine, internal medicine, cardiology, and endocrinology.

We selected patients aged 50 years or older with at least one CVD risk factor who attended for routine consultation with the participating physicians. The CVD risk factors comprising the inclusion criteria are described in table 3. Patients with a history of a previous CVD event, defined as myocardial infarction, angina (stable or unstable), stroke, or transient ischemic cerebrovascular event were excluded, as well as patients already participating in a clinical trial. All participating patients provided signed informed consent.

**Table 3:** Criteria to include patients in the EURIKA study

1. Age 50 years or older	
2. Free of clinical cardiovascular disease	
3. At least one of the following cardiovascular risk factors (as assessed from the most recent data in the clinical record or anthropometry for obesity)	
a) Dyslipidaemia	- LDL cholesterol ≥ 4.1 mmol/l (160 mg/dl), or
	- HDL cholesterol < 1.036 mmol/l (40 mg/dl) in men, and < 1.30 mmol/l (50 mg/dl) in women, or
	- Triglycerides ≥ 1.7 mmol/l (150 mg/dl), or
	- Under lipid-lowering medication
b) Hypertension	- Systolic blood pressure ≥ 140 mm Hg, or
	- Diastolic blood pressure ≥ 90 mm Hg, or
	- Under antihypertensive medication
c) Smoking	- Current or former smoker, with > 100 cigarettes smoked in lifetime
d) Diabetes mellitus	- Fasting plasma glucose ≥ 7.0 mmol/l (126 mg/dl), or
	- Under antidiabetic medication (insulin or oral medications)
e) Obesity	- Body mass index ≥ 30 kg/m^2^, or
	- Waist circumference ≥ 102 cm in men and ≥ 88 cm in women

Patients who met the selection criteria were randomly selected by the attending physician. The sample size is approximately 600 patients per country for a total study population of 7,641 patients. A sample size of 600 subjects allows for estimating the prevalence of risk factor control with a 95% confidence interval of 5% for an expected control prevalence of 50% (worst possible case) and a risk factor prevalence of 50%. Table 4 shows the participating patients by sex, age and country. The percentage of participating patients among those invited was 60.1% for the whole study (range 48.2% in Germany to 77.9% in Norway). Though patients were asked about reasons for not participating, in most cases they did not provide any or simply reported that they had no interest or time to participate.

**Table 4:** Patients participating in the EURIKA study, by sex, age and country

		Sex		Age	
			
	Total	Men	Women	< 65 years	≥ 65 years
	N	N (%)	N (%)	N (%)	N (%)
**Austria**	624	297 (47.6)	327 (52.4)	402 (64.4)	222 (35.6)
**Belgium**	638	312 (48.9)	326 (51.1)	342 (53.6)	296 (46.4)
**France**	593	325 (54.8)	268 (45.2)	329 (55.5)	264 (44.5)
**Germany**	678	333 (49.1)	345 (50.9)	309 (45.6)	369 (54.4)
**Greece**	620	285 (46.0)	335 (54.0)	380 (61.3)	240 (38.7)
**Norway**	611	298 (48.8)	313 (51.2)	373 (61.1)	238 (38.9)
**Russia**	604	192 (31.8)	412 (68.2)	492 (81.5)	112 (18.5)
**Spain**	642	330 (51.4)	312 (48.6)	383 (59.7)	259 (40.3)
**Sweden**	628	315 (50.2)	313 (49.8)	324 (51.6)	304 (48.4)
**Switzerland**	667	352 (52.8)	315 (47.2)	325 (48.7)	342 (51.3)
**Turkey**	663	313 (47.2)	350 (52.8)	511 (77.1)	152 (22.9)
**United Kingdom**	673	344 (51.1)	329 (48.9)	349 (51.9)	324 (48.1)
**Total**	7641	3696 (48.4)	3945 (51.6)	4519 (59.1)	3122 (40.9)

### Study variables and methods of data collection

Information was collected prospectively at both the physician and the patient level. A physician specific questionnaire captured information regarding physician demographics, practice settings, cardiovascular prevention beliefs and management. A patient-specific questionnaire captured information from clinical records and patient's interview, regarding sociodemographic data, CVD risk factors, current medications, comorbidity, and others aspects of CVD prevention and management Anthropometry and blood pressure readings were obtained under standardized conditions for each patient. Both physician and patient-level questionnaires were translated into local language.

A fasting blood sample was obtained on the same day as the outpatient consultation or, if this was not possible, on the following day. With the exception of Russia, where laboratory determinations were performed locally, the blood samples were sent to a central laboratory in Belgium (The Bio Analytical Research Corporation, http://www.barclab.com, Belgium) for assessment of serum lipids, apo AI, apo B, hs-CRP, uric acid, and creatinine. Additionally, serum samples were stored at -70°in a central biobank (BARCLAB, Gent, Belgium), only for future determination of emergent CVD biomarkers.

Table 5 includes a full description of data collection methods and study variables in the EURIKA study. In each country, a 10% random sample of all centres with participating physicians underwent a site visit for data monitoring and audit, to ensure data quality.

**Table 5:** Data collection methods and main study variables in EURIKA

Collection method	Variables
Questionnaire addressed to the physician	Academic training, work-setting and other characteristics of the physicians
	- Conditions in usual daily practice for cardiovascular risk factors measurement.
	- The use of global cardiovascular risk assessment and barriers encountered in clinical practice.
	- Knowledge, attitudes, and practices of physicians regarding cardiovascular prevention and management
	- For every patient, information on management of cardiovascular risk (frequency of cardiovascular risk factors assessment, lifestyle counselling, therapeutic goals, treatment adherence).
Questionnaire addressed to patients, and clinical record abstraction	Patients' demographic and psychosocial characteristics
	- Relevant family medical history: early cardiovascular event
	- CVD risk factors related to lifestyle (tobacco smoking, physical activity)
	- Comorbidity
	Current medication: antihypertensives, statins and other lipid-lowering drugs, oral antidiabetics, insulin, anticoagulants, aspirin, and combination drug therapy
Laboratory results taken from the clinical record (most recent blood data and physical examination during the previous year)	- Total cholesterol, LDL cholesterol, HDL cholesterol, non-HDL cholesterol, triglycerides, blood pressure, hemoglobine-A1c
Measurements performed on patients during the medical visit	Weight, height, waist and hip circumference, and blood pressure, under standardized conditions
Blood sample collected during the medical visit	- Total cholesterol, LDL cholesterol, HDL-cholesterol, non-HDL cholesterol, triglycerides, apolipoprotein B, apolipoprotein AI, hemoglobine-A1c, high-sensitivity C-reactive protein, creatinine, uric acid.

### Statistical analysis

The main analyses will be conducted according to a statistical plan drafted before completion of data collection. Statistical analyses will address the main study objectives. Accordingly, estimates of control of CVD risk factors will be provided for each country, by sex, age group (50-64 years, 65 years and older) and 10-year risk of CVD death (below 5%, 5% or more). The cut-points defining control of each risk factor will be taken from the European Guidelines on CVD prevention [4]; for estimates of dyslipidaemia control based on apo B and non-HDL cholesterol, we will use the cut-points agreed in the consensus statement from the American Diabetes Association and the American College of Cardiology Foundation [22]. The risk of CVD death will be calculated with the SCORE equation; for Belgium, France, Greece, Spain and Turkey using the SCORE equation for low-risk regions, while the equation for high-risk regions will be used for participants from Austria, Germany, Norway, Russia, Sweden, Switzerland and the United Kingdom [13]. Ancillary analyses might use SCORE equations calibrated with the national prevalence of CVD risk factors and CVD mortality rates in those countries where available, or country-specific CVD risk equations such as QRISK2 from the United kingdom[28].

The same type of descriptive statistical approach will be used for data on barriers faced by physicians in controlling CVD risk factors, as well as their knowledge of and attitudes toward CVD prevention.

## Discussion

We expect the EURIKA study to provide important information to quantify the degree of control of CVD risk factors and to identify barriers to successful CVD prevention in Europe, both at the physician and patient level. The transnational setting of EURIKA will also create opportunities for benchmarking good clinical practice across countries.

A potential limitation of the EURIKA study is the lack of a comprehensive framework for physician sampling in all European countries. We used the OneKey database, which is the largest available database of practicing physicians in Europe, although not statistically representative of all European physicians. As a further limitation, the participation rate among invited physicians was not optimal. It is possible that physicians more competent in CVD prevention were more likely to agree to participate; in this case, the results of EURIKA study could provide a best-case scenario that may slightly overestimate the control of CVD risk factors and quality of care in usual clinical practice. Nevertheless, the large number of practitioners included, the coverage of all relevant medical specialties and work-settings, and the random selection of study patients, suggest that the EURIKA study is likely to provide a comprehensive picture of the status of primary CVD prevention across Europe, which is as accurate as practically possible.

As additional strengths, the EURIKA study has used standardized procedures for data collection according to a common protocol for all countries. The questionnaire was easy to complete, and data abstracted from clinical records refer to basic and frequently assessed clinical variables, many of them from laboratory tests represented in the inclusion criteria. Also, blood samples have been analyzed with the same methods in a central laboratory, and a biobank has been set up for future studies. Most of the questions physicians were asked regarding knowledge and attitudes about CVD prevention were based on previously field-tested questionnaires. Moreover, as each physician had to report on only a few patients, questionnaire burden has been minimized. Lastly, assessment of control of CVD risk factors is based on objective measures (blood pressure readings, anthropometry, and laboratory results) specifically obtained for the EURIKA study. Thus, data quality in the EURIKA study is likely to exceed that of studies relying solely on data abstraction from clinical records and interviews.

## Competing interests

JP Halcox and J Dallongeville have received speaker fees and consulting fees from AstraZeneca. PG Steg reports receiving research grants from Servier; speaking or consulting for Astellas, AstraZeneca, Bayer, Boehringer-Ingelheim, Bristol-Myers Squibb, Daiichi-Sankyo, Endotis, Glaxo Smith Kline, Menarini, Medtronic, Merck-Sharpe-Dohme, Otsuka, Pierre Fabre,Roche, sanofi-aventis, Servier, The Medicines Company; and being a stockholder in Aterovax. R Hernández-Vecino, FJ Jiménez and EL Massó-González are employees of AstraZeneca. The rest of authors declare that they have no competing interests.

## Authors' contributions

FRA and JRB drafted the manuscript. All authors made substantial contributions to study protocol, reviewed the manuscript for important intellectual content, and approved the final manuscript.

## Pre-publication history

The pre-publication history for this paper can be accessed here:

http://www.biomedcentral.com/1471-2458/10/382/prepub
